# Limbic links to paranoia: increased resting-state functional connectivity between amygdala, hippocampus and orbitofrontal cortex in schizophrenia patients with paranoia

**DOI:** 10.1007/s00406-021-01337-w

**Published:** 2021-10-12

**Authors:** Sebastian Walther, Stephanie Lefebvre, Frauke Conring, Nicole Gangl, Niluja Nadesalingam, Danai Alexaki, Florian Wüthrich, Maximilian Rüter, Petra V. Viher, Andrea Federspiel, Roland Wiest, Katharina Stegmayer

**Affiliations:** 1 Translational Research Center, University Hospital of Psychiatry and Psychotherapy, University of Bern, Bern, Switzerland; 2 Institute of Diagnostic and Interventional Neuroradiology, Inselspital, University of Bern, Bern, Switzerland

**Keywords:** Paranoia, Schizophrenia, Resting-state fMRI, Functional connectivity, Limbic system, Amygdala

## Abstract

Paranoia is a frequent and highly distressing experience in psychosis. Models of paranoia suggest limbic circuit pathology. Here, we tested whether resting-state functional connectivity (rs-fc) in the limbic circuit was altered in schizophrenia patients with current paranoia. We collected MRI scans in 165 subjects including 89 patients with schizophrenia spectrum disorders (schizophrenia, schizoaffective disorder, brief psychotic disorder, schizophreniform disorder) and 76 healthy controls. Paranoia was assessed using a Positive And Negative Syndrome Scale composite score. We tested rs-fc between bilateral nucleus accumbens, hippocampus, amygdala and orbitofrontal cortex between groups and as a function of paranoia severity. Patients with paranoia had increased connectivity between hippocampus and amygdala compared to patients without paranoia. Likewise, paranoia severity was linked to increased connectivity between hippocampus and amygdala. Furthermore, paranoia was associated with increased connectivity between orbitofrontal and medial prefrontal cortex. In addition, patients with paranoia had increased functional connectivity within the frontal hubs of the default mode network compared to healthy controls. These results demonstrate that current paranoia is linked to aberrant connectivity within the core limbic circuit and prefrontal cortex reflecting amplified threat processing and impaired emotion regulation. Future studies will need to explore the association between limbic hyperactivity, paranoid ideation and perceived stress.

**Supplementary Information:**

The online version contains supplementary material available at 10.1007/s00406-021-01337-w.

## Introduction

Most people have a sense of security, i.e. feel generally safe in their social environment [1–3]. The perception of threat, e.g. being harmed or persecuted by others, is extremely distressing. Paranoia describes a spectrum of distressing experiences including mistrust, suspiciousness, ideas of reference, feelings of persecution, and delusions; the extreme end of this spectrum includes persecutory delusions that are held with great conviction [3]. While paranoia was conceptualized as a phenomenon of serious psychiatric disorders, research now demonstrates paranoid thinking in the general population with low severity in about one-quarter of the people, but high severity in up to 2% [4], maintained by a range of cognitive and affective factors (i.e. low self-esteem, worry, sleep dysfunction, perceptual anomalies, and reasoning biases) [3, 5]. Delusions with paranoid content are key features of schizophrenia spectrum disorders but also occur in other psychopathologies [6, 7]. Persecutory delusions are found in up to 70% of schizophrenia patients, cause significant distress, and trigger safety behaviors [8, 9]. Importantly, paranoid delusions have been found to lead to serious acts of violence in first-episode psychosis [9].

While researchers are beginning to unravel the cognitive and emotional factors of delusion formation across different diagnoses, a neurobiological model of paranoia is still missing. Paranoia is associated with negative self-beliefs, anxiety, and depression [2] and includes perceptions of immediate threat [3]. Given these negative emotions, the limbic system should play a critical role in the formation and perpetuation of paranoia, particularly the amygdala, hippocampus, and orbitofrontal cortex [10]. Indeed, evidence from structural and perfusion MRI studies in patients with paranoia seems to support this notion. For example, medial temporal lobe structures, particularly the amygdala, show structural alterations in patients with paranoia [11–13]. In addition, two studies reported increased resting-state perfusion of the left amygdala in paranoid patients with schizophrenia, arguing for increased neural activity [14, 15]. However, it remains unclear whether increased neural activity at rest in the amygdala was related to aberrant functional connectivity in the limbic system, and whether this aberrant connectivity was specific to experiences of paranoia.

In people at clinical high risk for psychosis, persecutory delusions were associated with increased functional connectivity from amygdala to the visual cortex [16]. But evidence on paranoia and functional connectivity in patients with schizophrenia is missing.

Several studies have explored the broader link between resting-state functional connectivity (rs-fc) and delusions generating complex and sometimes conflicting results. In schizophrenia, positive symptom severity, particularly of delusions and hallucinations was associated with rs-fc from thalamus to the middle temporal gyrus [17]. Likewise, effective connectivity at rest between thalamus and the anterior cingulate cortex correlated with positive symptom severity in schizophrenia, particularly the items delusions and persecution [18]. In contrast, functional connectivity between amygdala and precentral gyrus was decreased in first episode patients and linked to increased positive symptom severity, which may include persecutory delusions [19]. At network level, lower connectivity between and within resting-state networks including default mode network (DMN), salience network (SAL), sensorimotor network (SMN), or central executive network (CEN) was associated with severe positive symptoms in schizophrenia [20]. The limited evidence available suggests some association between delusions and alterations (both increases and decreases) in the cerebral resting state. However, the specific neural pattern associated with paranoia remains unknown.

While rs-fc from the amygdala to various brain regions declines with age in typical neurodevelopment, in youth with psychosis this development was deteriorated, leading to increased connectivity between amygdala and striatum, thalamus, ventrolateral prefrontal cortex and occipital cortex [21]. These findings suggest a lack of specialization during neural maturation in subjects with psychosis, which might give rise to increased resting-state activity in the limbic system and paranoid ideation.

In sum, while perfusion MRI studies suggest increased resting-state neural activity in the amygdala in patients with paranoia, we have currently little understanding of the functional connectivity within the limbic structures in patients with current paranoia. Particularly, the connectivity between amygdala, hippocampus, nucleus accumbens or orbitofrontal cortex requires elucidation.

Here, we aimed at exploring associations between aberrant neural activity at rest in the limbic system and current paranoia severity in subjects with schizophrenia. We hypothesized that patients with current paranoia would demonstrate increased functional connectivity at rest between key components of the limbic system when compared to patients without paranoia. In addition, we expect that connectivity within the limbic system was increased as a function of paranoia severity. Therefore, we tested these associations in both categorical and dimensional measures of paranoia.

## Subjects and methods

### Subjects

The current study included 89 patients with schizophrenia spectrum disorders according to DSM-5 criteria (schizophrenia, schizoaffective disorder, brief psychotic disorder, schizophreniform disorder) and 76 healthy controls (Table 1, Part A, Part C) from three different studies that used functional neuroimaging and comprehensive assessment of symptoms in patients with schizophrenia spectrum disorders [OCoPS-P (Overcoming Psychomotor Slowing in Psychosis) ClinicalTrials.gov Identifier: NCT03921450 [22] 10 patients, 17 controls; GNI (neural correlates of gesture deficits in schizophrenia) [23, 24] 46 patients, 44 controls; IPSS (interpersonal space study in schizophrenia) 30 patients, 14 controls]. We recruited patients at the in- and out-patient departments of the University Hospital of Psychiatry, Bern, and healthy controls via advertisement. Healthy controls were matched for age and gender. All participants provided written informed consent. The study protocols adhered to the declaration of Helsinki and were approved by the local ethics committee. General exclusion criteria for all subjects were substance abuse or dependence other than nicotine, history of neurologic disease, head trauma with concurrent loss of consciousness, or history of electroconvulsive treatment, and any MRI counter-indication. Additional exclusion criteria for controls were a history of any psychiatric disorder, as well as any first-degree relatives with schizophrenia or schizoaffective disorder.

**Table 1:** Demographic information

(A): Demographic information of the whole study population			
	Controls	Patients	Comparison
*N*	76	89	n/a
Age (years, mean ± sd)	36.9 ± 13.4	37.4 ± 12.1	*t*(152.5) = 0.24, *p* = 0.81
Gender	45 M	56 M	*χ*^2^ = 0.24, *p* = 0.6
Education (years, mean ± sd)	14.9 ± 3.1	13.2 ± 2.9	*t*(151.8) = 3.79, *p* < 0.001
Duration of illness (years, mean ± sd)	n/a	11.3 ± 11.2	n/a
Total PANSS (mean ± sd)	n/a	72.2 ± 16.3	n/a
Positive PANSS (mean ± sd)	n/a	17.7 ± 5.9	n/a
Negative PANSS (mean ± sd)	n/a	18.3 ± 6.00	n/a
Medication (OLZ eq.) (mean ± sd)	n/a	13.0 ± 9.7	n/a

(B): Demographic information of the schizophrenia population based on the paranoia score			
	Paranoia	Non-paranoia	Comparison
*N*	49	40	
Age	36.7 ± 12.5	38.3 ± 11.7	*t*(85.3) = 0.65, *p* = 0.52
Gender	32 M	24 M	*χ*^2^ = 0.27, *p* = 0.6
Duration of illness (years, mean ± sd)	11.3 ± 10.3	11.2 ± 11.7	*t*(65.38) = 0.03, *p* = 0.97
Medication	14.4 ± 9.3	12.1 ± 9.9	*t*(77.39) = 1.14, *p* = 0.26
Paranoia score	13.2 ± 3.0	6.9 ± 1.7	*t*(78.5) = 12.5, *p* < 0.001
P1	4.3 ± 1.6	2.1 ± 0.9	*t*(77.2) = 8.5, *p* < 0.001
P4	3.2 ± 1.0	1.9 ± 0.9	*t*(80.4) = 6.0, *p* < 0.001
P5	3.0 ± 1.6	1.4 ± 0.7	*t*(70.7) = 6.2, *p* < 0.001
P6	2.8 ± 1.3	1.5 ± 0.8	*t*(80.7) = 5.6, *p* < 0.001

(C): Comparison across the three groups						
	*Df*		*F* value		Pr(> *F*)	
Age	2		0.22		0.8027	
Years of education	2		7.381		0.0008623	

Post hoc lsmeans (Years of education)					
Contrast	Estimate	SE	*df*	*t*.ratio	*p* value
Control—(non-paranoia)	1.99	0.584	158	3.404	0.0024
Control—paranoia	1.62	0.558	158	2.897	0.0119
(Non-paranoia)—paranoia	− 0.37	0.644	158	− 0.574	0.8344
Gender chi-square across the 3 groups *χ*^2^ = 0.49, *p* = 0.77					

*sd* standard deviation, *PANSS* Positive And Negative Syndrome Scale, *CPZ eq.* chlorpromazine-equivalent (mg/day), *M* male, *χ*^2^ chi-square

We assessed the current psychopathology with the Positive And Negative Syndrome Scale (PANSS) [25]. In addition, we assessed symptom history using the Comprehensive Assessment of Symptoms and History (CASH) [26]. All patients were on antipsychotic medication (including thioxanthenes (*n* = 27), olanzapine (*n* = 21), clozapine (*n* = 11), haloperidol (*n* = 10), quietiapine (*n* = 6), amisulpride (*n* = 5), chlorpromazine (*n* = 2), lurasidone (*n* = 2)). Due to the large variability in the medication type, we calculated the mean olanzapine equivalents (OL eq.) according to Leucht [27] (Table 1, Part A). We performed psychopathology assessments within 48 h of MRI scanning.

### Paranoia assessment

Multiple studies evaluated paranoia based on different PANSS scores. In the current study, to explore all the possible facets of paranoia, we used a modified version of the composite score established by Williams and collaborators in 2004 [28]. This paranoia score consists of the sum of four items of the PANSS (P1: Delusions, P4: excitement, P5: Grandiosity and P6: Suspiciousness and persecution), ranging 4–28. We used this score in two types of analyses: (1) group classification and (2) using the composite paranoia score as a continuous variable.

For the group classification analysis, we compared the rs-fMRI connectivity between “Patients with paranoia” and “Patients without paranoia”. To classify the patients, we applied a median split, i.e. a composite paranoia score of 10 (Supplement 1.A, Table 1, Part B). Accordingly, patients with paranoia had a score ≥ 10, while those without paranoia had a score of < 10. Subsequently, the two groups were compared on neuroimaging measures. The PANSS score on each of the 4 items was significantly higher in the patients with paranoia compared to the Patients without paranoia (Supplement 1.B). Additionally, we performed correlations between the composite paranoia score and the positive and negative subscore of the PANSS, which are presented in Supplement 1.C. An alternative approach often used in the literature is to aggregate patients according to the PANSS item P6 (suspiciousness) [15]. Accordingly, we repeated the analyses based on the P6 item score, using a cut-off for the group classification (patients with paranoia: P6 score ≥ 3) (see Supplement 3A).

For the continuous variable analysis, we evaluated the rs-fMRI connectivity associated with the composite paranoia score. We referred to this analysis as severity analysis.

### MRI acquisition and preprocessing

#### Acquisition

Subjects lay horizontally in the MR scanner and their arms rested beside their trunk. To reduce head motion, foam pads were placed around the participants’ head and we explicitly instructed participants to avoid head motion.

For 43 patients and 32 healthy controls, the MRI scans were acquired on a 3 T Prisma MRI scanner (Siemens, Germany). The MRI sequences acquired included the following: one T1-weighted MPRAGE scan (8 min 22 s covering 176 sagittal slices, 1 mm thick, TR = 5000 ms, TE = 2.98 ms, flip angle 1 = 4°, flip angle 2 = 5°, voxel size = 1 × 1 × 1 mm), and one rs-fMRI sequence using a multi-band echoplanar 2D (10 min and 11 s covering 600 volumes of 72 slices; 2.5 mm thick, TR = 1000 ms, TE = 37 ms, flip angle = 30°, voxel size = 2.5 × 2.5 × 2.5 mm FOV = 230 × 230 mm, GRAPPA = 1, multiband acceleration factor = 8).

For 46 patients and 44 healthy controls, the MRI scans were acquired on a 3 T TrioTim MRI scanner (Siemens, Germany). The MRI sequences acquired included the following: T1-weighted MDEFT scan (13 min 43 s covering 176 sagittal slices, 1 mm thick, TR = 7.92 ms, TE = 2.48 ms, flip angle = 16°, voxel size 1 × 1 × 1 mm), and one rs-fMRI sequence using a single-band echoplanar 2D (8 min and 40 s covering 256 volumes of 38 slices; TR = 2000 ms, TE = 30 ms, flip angle = 90°, voxel size = 3.6 × 3.6 × 3.0 mm, FOV = 230 × 230 mm, GRAPPA = 2).

#### Resting-state fMRI pre-processing

We analyzed the rs-fMRI data using SPM12 (https://www.fil.ion.ucl.ac.uk/spm/software/spm12) and the CONN toolbox v19 (functional connectivity toolbox; http://www.nitrc.org/projects/conn).

The preprocessing steps included slice-timing correction, motion realignment, co-registration between the functional scans and 3D-T1w scans of each participant and normalization of the functional scans to MNI space using DARTEL and smoothing using a Gaussian filter of 6 mm. In addition, we defined thresholds for participants exclusion due to excessive head motion according to the literature [29–32]: absolute head motion involved translation greater than 2 mm or rotation greater than 2° (See Supplement 5 A), or if mean frame-wise displacement (FD) (calculated as defined by Power et al. [33]) was greater than 0.5 mm. Using these thresholds resulted in no exclusion of any participants. Furthermore, none of the participants was excluded due to large displacements or spikes in any of the six directions (See Supplement 5). Moreover, one-way ANOVA did not reveal any significant differences in the mean FD between the three groups (patients with paranoia, patients without paranoia and healthy controls, *p* = 0.352, See Supplement 5 B). We included the mean FD of each participant as a covariate of non-interest in further analyses. We also performed a denoising step using noise component of white matter and cerebrospinal fluid tissues and motion parameters (12 potential noise components defined from the estimated subject‐motion parameters to minimize motion‐related BOLD variability, three translation and three rotation parameters plus their associated first‐order derivatives) as regressors, a despiking procedure, a linear detrending and band-pass filtering (0.01–0.1 Hz).

#### Region of interest definition

We defined eight regions of interest (ROIs): the bilateral amygdala, hippocampus, nucleus accumbens, and orbitofrontal cortex (OFC). These ROIs were selected from the WFU PickAtlas [34, 35]. The exact coordinates of the center of each ROI and its size were as follows: left amygdala (*x* = −24, *y* = −2, *z* = −19; size: 1760 mm^3^), right amygdala (*x* = 27, *y* = −1, *z* = −19; size: 1984 mm^3^), left hippocampus (*x* = −30, *y* = −22, *z* = −14; size: 1112 mm^3^), right hippocampus (*x* = 30, *y* = −22, *z* = −14; size: 976 mm^3^), left nucleus accumbens (*x* = −14, *y* = 8, *z* = −12; size: 984 mm^3^), right nucleus accumbens (*x* = 12, *y* = 10, *z* = − 12; size: 984 mm^3^), and left OFC (*x* = −17, *y* = 46, *z* = −15; size: 7704 mm^3^), right OFC (*x* = 18, *y* = 47, *z* = −16; size: 7976 mm^3^) (see Supplement 2).

#### Intracranial volume (ICV) calculation

Structural brain alterations have been extensively described in schizophrenia [36]. Accordingly, to correct for potential inter-individual head size differences, we used the intracranial volume (ICV) as a covariate of non-interest. The volumes extracted from the segmentation (white matter, grey matter, and cerebrospinal fluid) were summed up to provide an estimate of ICV for each participant. 

#### Resting-state fMRI analysis

To measure functional changes in rs-fc, we compared the functional connectivity at the region of interest (ROI) level using both ROI-to-ROI and ROI-to-voxels analyses and at the whole-brain level using independent component analysis (ICA, see Supplement 4A).

#### Region of interest analyses

We performed two types of ROI analyses: ROI-to-ROI and ROI-to-voxels to compare the three groups: healthy controls, patients with and without paranoia. In the patients' population, we also correlated the rs-fc with the severity of paranoia. In ROI-to-ROI analyses, we aimed to explore rs-fc between the eight ROIs, which are supposed to be strongly interconnected and involved in the neural dysfunction associated with paranoia. We analyzed 28 connections among the 8 ROIs and reported cluster-based inferences [37]. To test the influence of the scanner on the rs-fc, we compared the rs-fc of the healthy controls and of the patients between the two scanner types and added scanner type as covariate of non-interest. To test whether neural activity in these ROIs is linked to neural activity throughout the rest of the brain, we also performed ROI-to-voxels analyses. To compare rs-fc between the three groups: healthy controls, patients with and without paranoia, we performed an ANCOVA including scanner type, age, years of education, mean FD, and ICV as covariates of non-interest. For between patients’ comparisons, we used scanner type, mean FD, age, medication dosage and ICV as covariates of non-interest. In addition, to evaluate positive and negative correlations between rs-fc and the severity of paranoia, we used the paranoia score as covariate of interest and scanner type, age, medication dosage and ICV as covariates of non-interest.

In addition, to evaluate any difference in the rs-fc between patients with schizophrenia and healthy controls that might appear outside the ROIs network, we used whole-brain independent component analysis (ICA), the details of this procedure are presented in the supplement (Supplement 4 A).

### Statistics

For demographics analyses, we used R to perform a Student’s t test to compare the age and education between healthy controls and patients with schizophrenia, and a Chi-square repartition test to compare the gender distribution between healthy controls and patients with schizophrenia. Finally, we also performed an ANCOVA to compare age and education between the three groups and a Chi-square repartition test to compare the gender distribution between the three groups. A *p* value < 0.05 was considered statistically significant. For ROI-to-ROI analyses, we set the significant threshold as a cluster threshold p value qFDR corrected < 0.05. For ROI-to-voxels analyses, we set a cluster-forming threshold of *p* = 0.005 and a p value qFDR corrected < 0.05 for the cluster-wise threshold.

In addition, in the supplement, all of these analyses are presented using the P6 item cut-off instead of the composite paranoia score (see Supplement 3 B).

## Results

No significant differences were observed in the rs-fc between two scanners acquisition neither in healthy subjects nor in patients (Supplementary Fig. 5). [Healthy subjects: no significant difference between the two scanners: *n* = 76, *T*(74), connection threshold of *p* = 0.001 and cluster threshold *p* value qFDR corrected < 0.05. Paranoia patients: no significant difference between the two scanners: *n* = 57, *T*(55), connection threshold of *p* = 0.001 and cluster threshold *p* value qFDR corrected < 0.05. Non-paranoia patients: no significant difference between the two scanners: *n* = 32, *T*(30), connection threshold of *p* = 0.001 and cluster threshold *p* value qFDR corrected < 0.05]. Accordingly, the data sets from the two scanners were merged and the scanner type was used as a covariate of non-interest in the following analyses.

### ROI-to-ROI analysis between the three groups

**Fig. 1:**
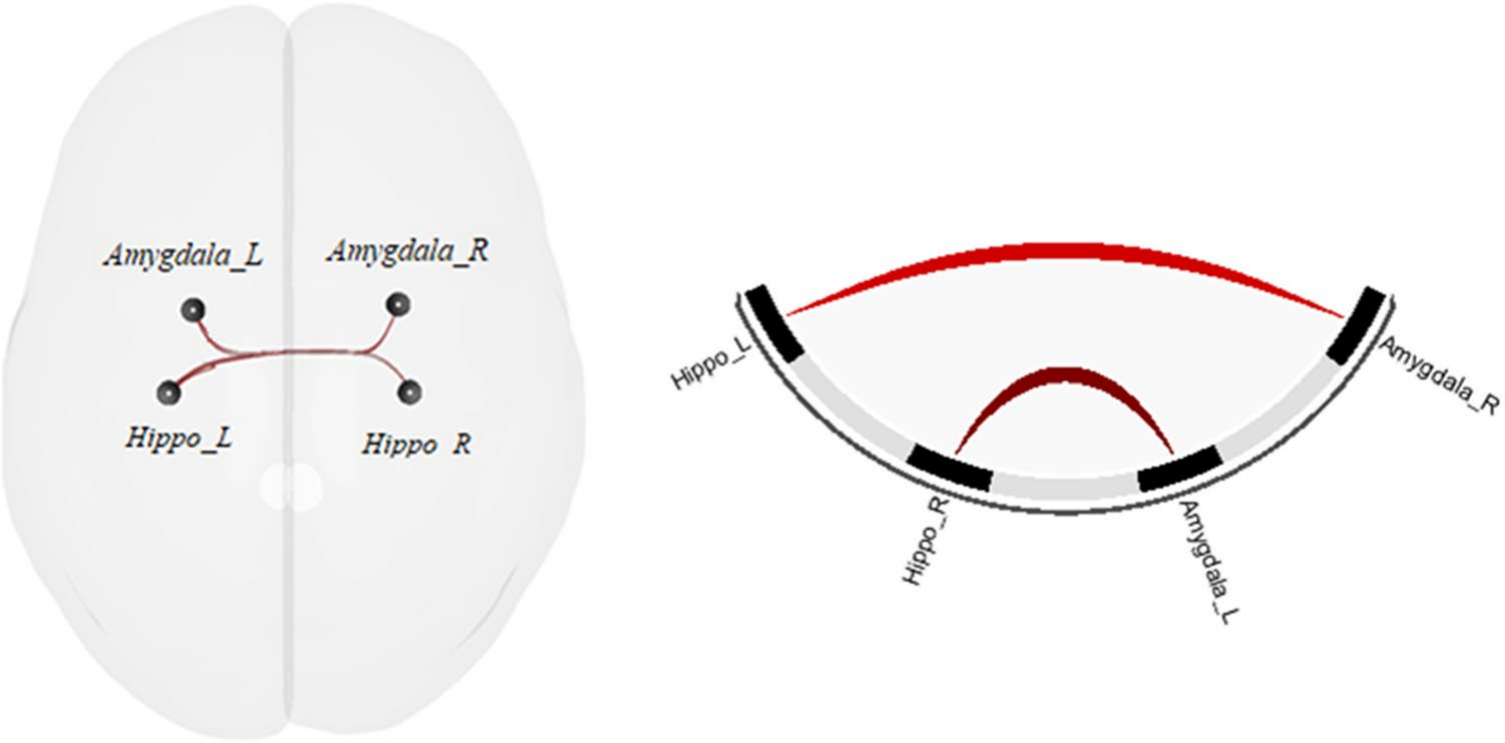
ROI-to-ROI rs-fc differences between paranoia and non-paranoia groups. *Hippo* hippocampus, *R* right, *L* left

**Fig. 2:**
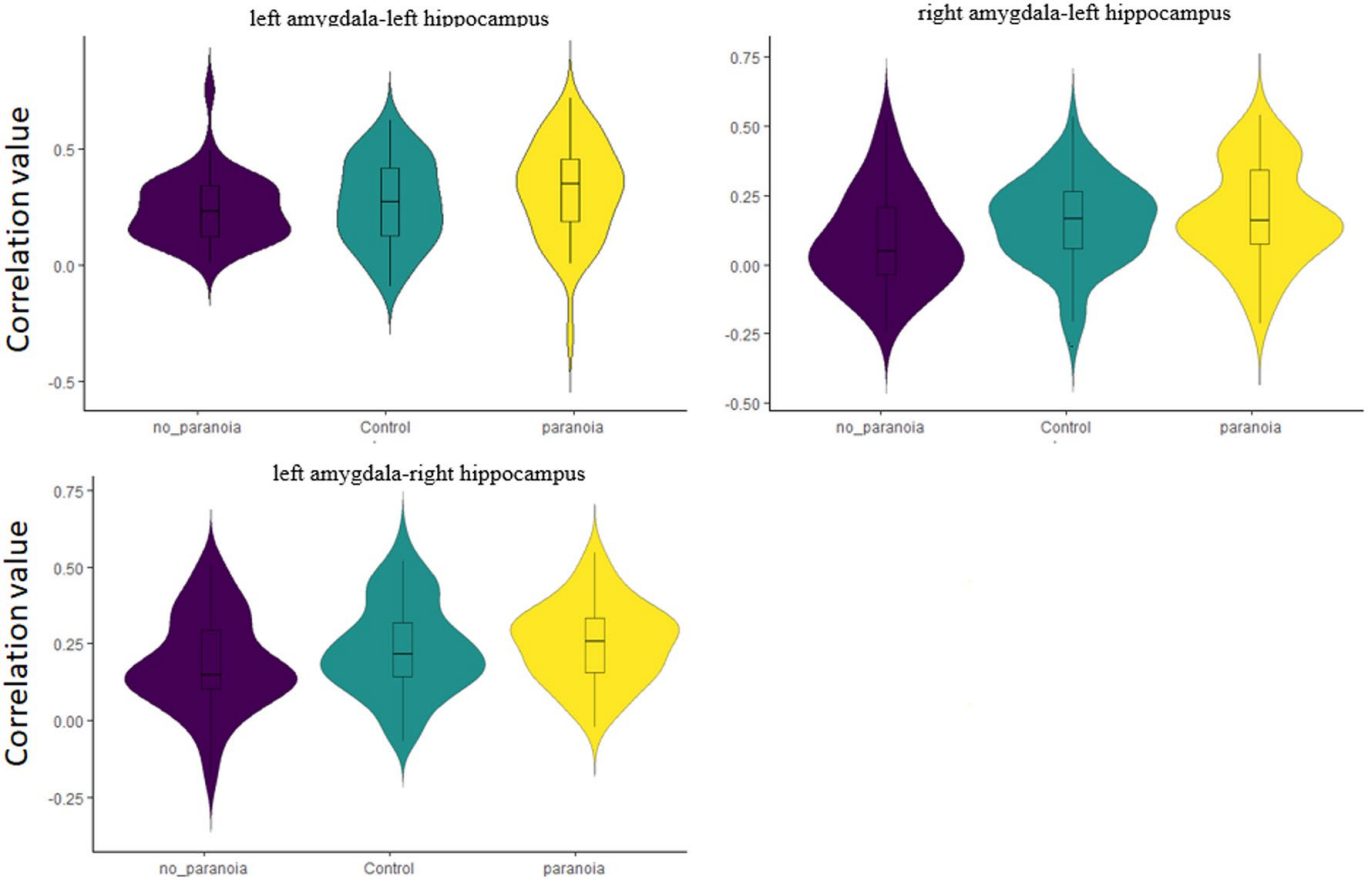
Violin distributions of the ROI-to-ROI correlation value for each subgroup in the pairs of interest. In each violin, a box plot is added. The center line represents the median value, the lower bound of the box represents the 25th percentile, the upper bound of the box the 75th percentile, and the whiskers represent three times the interquartile range

The ANCOVA comparing healthy controls, patients with and patients without paranoia demonstrated a significant group effect (*F*(8,308) = 2.55, *p*_qFDR_ = 0.049). The post-hoc t-tests revealed an increase of the rs-fc between the hippocampus and the amygdala in the paranoia group compared to the non-paranoia group (Fig. 1, Table 2, Part A). The differences between the control and the two patient groups are not significant. To depict this lack of difference with the healthy controls, we plotted the connectivity values for each ROI-pair with significant group difference across all groups (Fig. 2). Interestingly, healthy controls seem to have an intermediate position in terms of connectivity strength between patients with paranoia and patients without paranoia.

**Table 2:** Connectivity analyses

	ROI to ROI connection				Statistics		p-qFDR	
A	ROI-to-ROI rs-fc differences between the 3 groups							
	Group effect				F(8,310) = 2.59		0.48	
	Post hoc analyses							
	Paranoia vs healthy control groups ø							
	Non-paranoia vs healthy control groups ø							
	Paranoia vs non-paranoia groups				*F*(3,81) = 4.84		0.022	
	Hippo_R-Amygdala_L				*T*(81) = 2.35 *T*(81) = 2.01		0.021 0.047	
	Hippo_L-Amygdala_R							
B	ROI-to-ROI rs-fc changes with paranoia severity				*F*(3,81) = 7.10		0.001	
	Hippo_R -Amygdala_R Hippo_R -Amygdala_L Hippo_L -Amygdala_L Hippo_L -Amygdala_R				*T*(83) = 3.26 *T*(83) = 3.06 *T*(83) = 2.29 *T*(83) = 2.29		0.001 0.003 0.029 0.029	

	ROI	Cluster peak (*x, y, z*)	BA	Size		Cluster p-FWE		Cluster p-qFDR
C	ROI-to-voxels rs-fc differences between the 3 groups							
	Group effect ø							
	Exploratory analysis paranoia vs non-paranoia groups							
	Left sup OFC	− 50 + 28 − 08	BA 47	240		0.003		0.002
	Right sup OFC	−38 −71 + 48	BA 39	252		0.002		0.002
D	ROI-to-voxels rs-fc changes with paranoia severity							
	Left sup OFC	+ 36 + 24 −16	BA 187	94		0.016		0.015
	Left sup OFC	−46 + 22 −18	BA 156	94		0.046		0.021

*BA* broadman area, *hippo* hippocampus, *OFC* orbitofrontal cortex, *R* right, *L* left

### ROI-to-voxels analysis between the three groups

The ANCOVAs comparing rs-fc between healthy controls, patients with and patients without paranoia demonstrated no significant group effect for any of the seeds.

Due to the intermediate position of the healthy control group in terms of rs-fc (see previous analysis), we performed an additional exploratory analysis comparing only patients with and without paranoia using age, education, scanner type, medication dosage, and ICV as covariates of non-interest. We found an increase of the rs-fc in paranoia patients compared to patients without paranoia between the right OFC and the left BA 39 and between the left OFC and the left BA 47 (Table 2, Part B).

### ROI-to-ROI association with paranoia severity

**Fig. 3:**
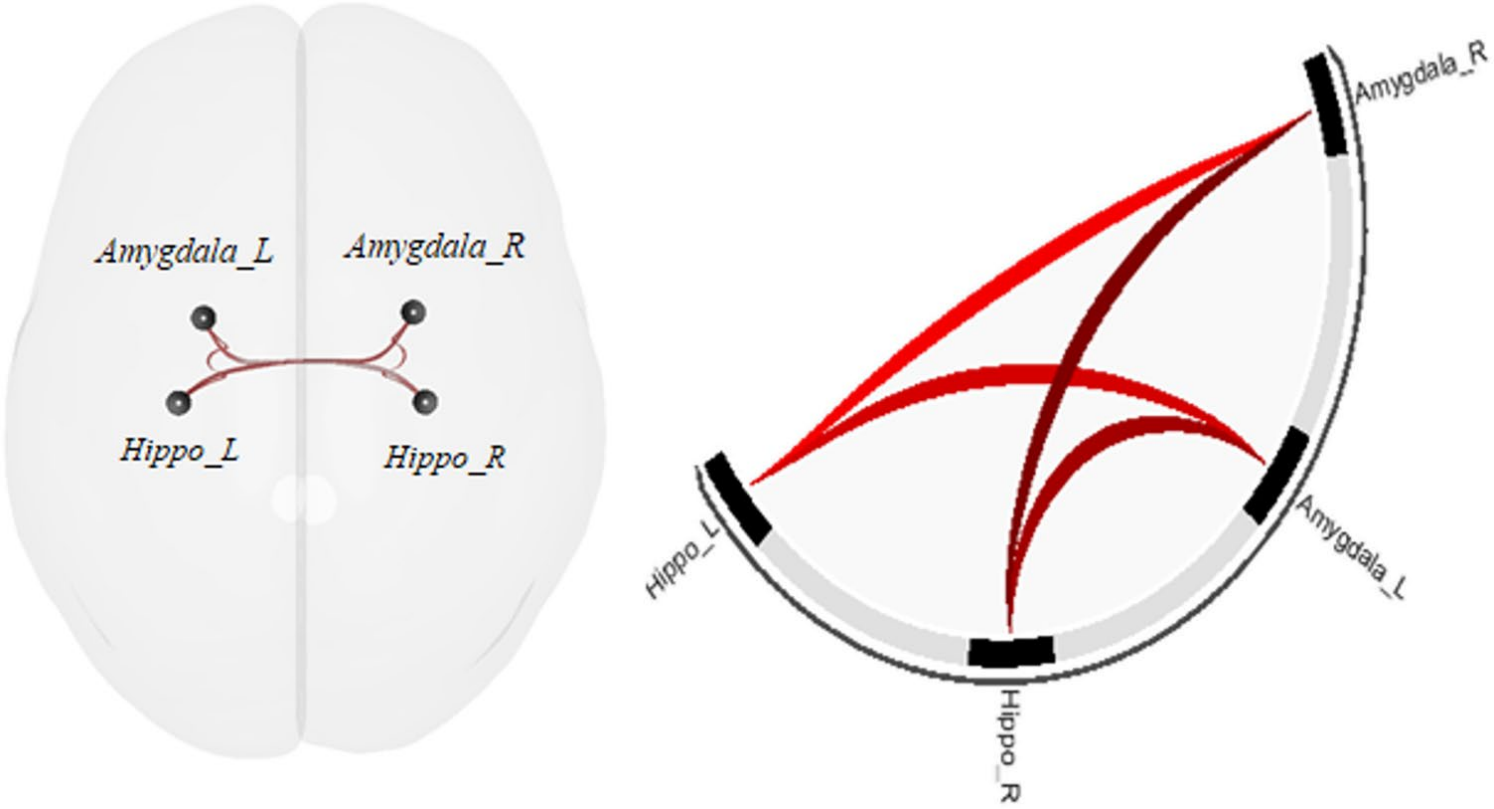
ROI-to-ROI rs-fc changes with paranoia severity maps. *Hippo* hippocampus, *R* right, *L* left

The ANCOVA exploring the impact of dimensional paranoia ratings on rs-fc on the 28 connections between the 8 ROIs demonstrated significant effects (*F*(3,81) = 7.10, *p*_qFDR_ = 0.0016). The post hoc t tests revealed that an increase in paranoia is associated with increased rs-fc between bilateral hippocampus and bilateral amygdala (Fig. 3, Table 2, Part C).

### ROI-to-voxels association with paranoia severity

The association between paranoia severity and the rs-fc connectivity between every ROI and the rest of the brain was explored. We observed a positive association between the left OFC and the bilateral BA 47 (Table 2, Part D), suggesting that increased paranoia was linked to stronger rs-fc between left OFC and bilateral BA 47.

### Additional exploratory analyses

In the supplementary material, we also describe results for an alternative classification of paranoia using the PANSS P6 item score. When patient groups were categorized according to the P6 item of the PANSS, 12 patients had paranoia and 77 had no paranoia. Due to the imbalance between the two groups, we did not perform the group comparison. Instead, we analyzed the effect of paranoia severity according to P6 on rs-fc, which demonstrated associations between amygdala or hippocampus and temporal cortex (see Supplement 3).

As no significant differences were observed in the rs-fc of the selected ROIs, we performed an additional analysis at the whole-brain level. A whole-brain ICA was used to compare rs-fc between patients with schizophrenia and healthy controls. We identified a reduced functional connectivity in patients compared to controls in the DMN and dorsal attention network (DAN) (Supplement 4B). Likewise, whole-brain ICA of rs-fc indicated multiple DMN alterations in patients with paranoia vs. healthy controls: reduction in the posterior cingulate, but increases in bilateral medial and dorsolateral prefrontal cortex, left angular gyrus, and left middle temporal gyrus (Supplement 4C).

## Discussion

Here, we tested rs-fc within the limbic system in schizophrenia patients with current paranoia. Probing rs-fc between bilateral amygdala, hippocampus, nucleus accumbens, and OFC, we found increased rs-fc between hippocampus and amygdala in patients with paranoia compared to patients without paranoia. This increase in rs-fc between bilateral hippocampus and amygdala was also linked to the severity of paranoia. In additional seed-to-voxel analyses, we found the left OFC to be connected to right BA47 in patients with paranoia. Finally, patients with paranoia had increased functional connectivity in prefrontal areas of the DMN when compared to healthy controls. Group differences in rs-fc were corroborated by regression analyses with dimensional assessments of paranoia.

The experience of paranoia is very unpleasant and stressful [3]. Patients engage in multiple safety behaviors when feeling distressed by paranoid ideation [8]. In addition, stress may also trigger further increases in paranoia in both patients with schizophrenia and in subjects vulnerable to psychosis [38–40]. Collectively, these negative emotions are very likely associated with increased neural activity in the limbic system. Indeed, two perfusion MRI studies demonstrated increased resting-state activity of the amygdala in paranoid patients with schizophrenia [14, 15]. Increased resting-state perfusion indicates stronger metabolic activity. In the resting-state, cerebral perfusion and functional connectivity are highly correlated particularly in the DMN and prefrontal cortex, while this correlation strength increases with task demands [41]. Thus, we could expect increased rs-fc from the amygdala in patients with current paranoia.

Threat processing engages ventral hippocampus, amygdala, and orbitofrontal cortex in fMRI tasks in healthy subjects [42–44]. Exposure to threat activated the amygdala for example during a computer gaming predator–prey task [40], while watching short video clips of threatening action [41], and engaged anterior hippocampus during an approach-avoidance conflict under varying levels of potential threat [39]. Likewise, testing patients with selective hippocampal lesions show altered avoidance behavior and threat processing [39]. In addition, the nucleus accumbens is important for processing avoidance [45]. Finally, the ventromedial and dorsolateral prefrontal cortices are critical in regulating the activity of this limbic network [46, 47].

Increased rs-fc between components of the subcortical amygdala pathway (hippocampus–amygdala) in paranoia suggests that this pathway is particularly amplified in paranoia, which might be critical to experience stress upon threat. This over-excitation could result from chronic neural alterations in the pathway, as suggested by converging animal models and neuroimaging studies. Indeed, rodent models of schizophrenia consider the ventral hippocampus to be particularly vulnerable to threat induced stress, which is probably due to the distinct maturational profile of fast-spiking parvalbumin interneurons (PIV) [48–50]. If threat-induced stress was sufficiently strong, it may trigger hippocampal PIV pathology including cell death [48]. To have this impact, threat either has to be extremely severe or needs to be experienced as extraordinary severe (e.g. in paranoia). Various stressors may activate glutamatergic projections from the amygdala to the hippocampal PIV in schizophrenia; strong stressors interact directly while mild stressors may exert the effect when amygdala regulation from prefrontal cortex is compromised [48]. Along this line, dysfunctional parvalbumin containing inhibitory interneurons seems to drive hippocampal hypermetabolism in schizophrenia [51]. Indeed, increased perfusion as an indicator of hypermetabolism as well as concurrent atrophy have been reported in the hippocampus in early psychosis [52–55] and subjects at risk [56], in which social stress may particularly attenuate positive symptoms [57]. Collectively, our functional connectivity findings are in line with studies demonstrating increased neural activity in the amygdala in patients experiencing paranoia [14, 15]. In addition, findings extend previous knowledge in demonstrating increased connectivity in key components of the limbic system compared to patients without paranoia. Furthermore, we may speculate that the experience of paranoia might also be linked to inefficient stress regulation. However, this proposed link between stress, limbic system hyperactivity, and paranoia requires further work to elucidate the association.

Outside the core limbic circuit, paranoia was also associated with increased rs-fc within the orbital and medial prefrontal cortex (OMPFC including the OFC and BA 47) and between the OFC and the parietal cortex (BA 39, angular gyrus). In general, the OMPFC constitutes a substantial portion of the cerebral cortex in primates. Although its function is not yet fully understood, studies in both non-human primates and humans suggest a role in decision-making and guidance of emotional and reward‐related behaviours [58]. Furthermore, BA47 is particularly involved in reappraising negative emotions and suppressing subsequent behaviors [59]. Thus, altered connectivity of the OMPFC in paranoia may either reflect cognitive threat processing and delusion formation, as OMPFC interacts with ventral hippocampus, basal amygdala, and medial prefrontal cortex. A task-based fMRI study argues for a role in cognitive threat processing [60]. When healthy subjects found threatening verbal statements to relate to themselves BA47 was active, while attention to threatening statements was linked to BA39 activity. Thus, both brain regions could be relevant for the formation of paranoid delusions. In line with these findings, paranoia severity was associated with rs-fc increases between OFC and BA47 in our study. However, as we investigated the cerebral resting state, we can only speculate whether increased connectivity suggests aberrant stress regulation, delusion formation or both. Therapeutic efforts may aim at reducing neural hyperconnectivity and hyperactivity in the limbic system.

The experience of paranoia may trigger safety behaviors, such as avoidance, which has been linked to Ncl. accumbens function [8, 45, 61]. We reported previously that structural connectivity between amygdala and Ncl. accumbens was associated with the severity of positive symptoms in schizophrenia [11]. Likewise, grandiosity and paranoid threat were linked to structural alterations in the pathway from the ventral tegmentum that includes Ncl. accumbens [12]. However, the Ncl. accumbens was not related to the presence or severity of paranoia using rs-fc. We may speculate that rs-fc may better reflect the experience of stress and threat in paranoia than paranoia-related avoidance.

Aberrant rs-fc in the DMN has become a frequent finding in schizophrenia. However, the direction of change and the DMN associations with schizophrenia symptoms remain subject of debate [62, 63]. DMN activity has been explained with self-referential processing, emotion regulation, and recollection of prior experiences [64]. We may speculate that self-referential processes and emotion regulation within the frontal DMN hubs are both critically involved in sustaining delusional threat beliefs that feed paranoia. Our findings in patients with current paranoia are in line with previous reports, demonstrating a correlation of frontal DMN connectivity at rest with positive symptoms, i.e. delusions and hallucinations [63, 65, 66]. We detected increased rs-fc in prefrontal cortex components of the DMN in paranoia patients compared to healthy controls. Likewise, our finding of reduced DMN connectivity at rest in schizophrenia compared to controls is in line with a meta-analysis and parts of the literature [65–67]. While altered connectivity within the DMN may not explain symptoms conclusively, it’s interaction with other cerebral networks is suggested to contribute to the clinical phenomena in schizophrenia [62, 68–70].

In a broader context, specific behaviors in schizophrenia are linked to patterns of brain alterations including grey matter reductions, increased resting-state neural activity as well as increased structural and functional connectivity within the key networks. In fact, in schizophrenia patients with psychomotor inhibition, we found reduced grey matter volume and increased resting-state perfusion in the supplementary motor area across samples, who in turn demonstrated increased rs-fc and structural connectivity with other motor areas of the brain [71–75]. The brain alterations in patients with paranoia follow a similar pattern: reduced grey matter volumes in amygdala and ventral striatum [13, 76], increased resting-state perfusion of the amygdala [14, 15], and increased rs-fc within a limbic network. Thus, specific alterations of cerebral circuit physiology may give rise to distinct symptoms in psychosis.

The most pronounced differences were noted between patients with and without paranoia. In the ROI-to-ROI or ROI-to-voxel analyses we failed to find differences between patients and controls, while we found differences at the network level (e.g. DMN). This pattern of findings argues for enormous biological variance between patients with schizophrenia that is even exceeding the differences between patients and controls, which is in line with larger analyses of structural brain changes in schizophrenia [77]. Therefore, our findings support a dimensional view on schizophrenia pathology that takes symptom dimensions into account. In the present study, we explored paranoia severity using a composite score based on 4 selected PANSS items (delusions, excitement, grandiosity and suspiciousness and persecution) as in previous studies [28]. As depicted in Supplentary 1C, the composite paranoia score correlates with the PANSS positive subscore but does not correlate with the PANSS negative score, which is in line with the idea that paranoia contributes to positive psychotic symptoms [78, 79].

### Limitations

The findings of the current study should be interpreted in light of some limitations. First, data of three studies in psychosis were pooled for this analysis. These data were acquired with two different 3 T Siemens scanners at Bern University. The differences in the acquisition parameters between the two scanners are mostly observed on the rs-fMRI sequences. The two sequences presented a different type of acquisition (single vs multiband), TR, volume numbers and voxel sizes. Even if some of these differences are corrected after preprocessing and first-level analysis, one could argue that the difference in acquisition might lead to a difference in the observed functional connectivity. However, in this study, the rs-fMRI analysis accounted for the acquisition differences between scanners, and the results remained unchanged after correction. Moreover, the group comparisons between the two scanners demonstrated no significant difference. One additional way to unambiguously check the inter-scanner reliability would be to perform the analyses separately for each acquisition type. However, due to the strong variability in the paranoiac symptoms and severity, large sample sizes are needed to catch the whole picture. In future studies, a more homogenous design and imaging acquisition parameters would be needed to confirm the present results. Second, despite the similarities between the three studies, specific rating scales for the assessment of paranoia have not been consistently applied. Specific scales on paranoia, either expert-rated or self-report are likely to be more sensitive in assessing paranoia severity than the PANSS score [28]. Third, patients were on antipsychotic medication, potentially influencing both paranoia severity and rs-fc. Moreover, the patients were all on different combinations of antipsychotic medication. Even if we used an equivalence scale (olanzapine equivalent) to evaluate the medication dosage of each patient and controlled for it in our statistical models, it is worth noting that equivalence scales can not depict the entire medication profile of the patients. With new medication coming, several antipsychotic drugs are missing from the equivalence scales and the establishment of these scales also suffers from some limitations such as the lack of very large randomized controlled trials to define dose equivalencies. Fourth, we focused on subjects with clear paranoia, neglecting the transdiagnostic similarities or subclinical paranoid experiences in the general population. Future studies should therefore test the associations of functional connectivity with dimensional measures of paranoia to capitalize on the whole spectrum of the paranoia continuum. Fifth, other studies on paranoia have included subjects with stronger symptom severity [13, 15]. But our sample was heterogeneous in terms of paranoia severity, thus particularly suited for dimensional assessments. In fact, the results of the regression analyses closely aligned with those of the group comparisons. Sixth, in the present study we focused exclusively on functional connectivity, as more homogenous population and design would be needed to test effective connectivity in the limbic system in paranoia. However, the present results provide a solid background for future studies. Seventh, our study included 89 patients with schizophrenia spectrum disorders (schizophrenia, schizoaffective disorder, brief psychotic disorder, schizophreniform disorder), but was not designed to analyse these groups of patients separately. Thus our results do not necessarily generalize to all schizophrenia spectrum disorders. Finally, we had no physiological data documenting stress in subjects with paranoia. Future investigations will allow integrating physiology with self-reported paranoia and brain imaging.

## Conclusion

The current study investigated rs-fc in patients with paranoia. Increased connectivity between hippocampus and amygdala as well as alterations in the DMN in patients with paranoia argue for dysfunctional threat processing in these patients. Instead of indicating a general phenomenon, these alterations in functional connectivity are specific to schizophrenia patients experiencing paranoia.

## Supplementary Information

Below is the link to the electronic supplementary material.

Supplementary file1 (DOCX 7561 kb)

## Data Availability

The statistical maps that support the findings of this study are available from the corresponding author upon reasonable request.
