# Supplementary material for: Limbic links to paranoia: increased resting-state functional connectivity between amygdala, hippocampus and orbitofrontal cortex in schizophrenia patients with paranoia
Source: Eur Arch Psychiatry Clin Neurosci. 2021 Oct 12;272(6):1021–32. doi: 10.1007/s00406-021-01337-w (PMC9388427; doi:10.1007/s00406-021-01337-w)
Supplement: Supplementary file 1 — Supplementary file1 (DOCX 7561 kb) [file 406_2021_1337_MOESM1_ESM.docx]

**Supplementary files**

**Supplement 1:
A.** Distribution of the composite paranoia score in the 89 patients. (Note that the score may range 4-28)

**
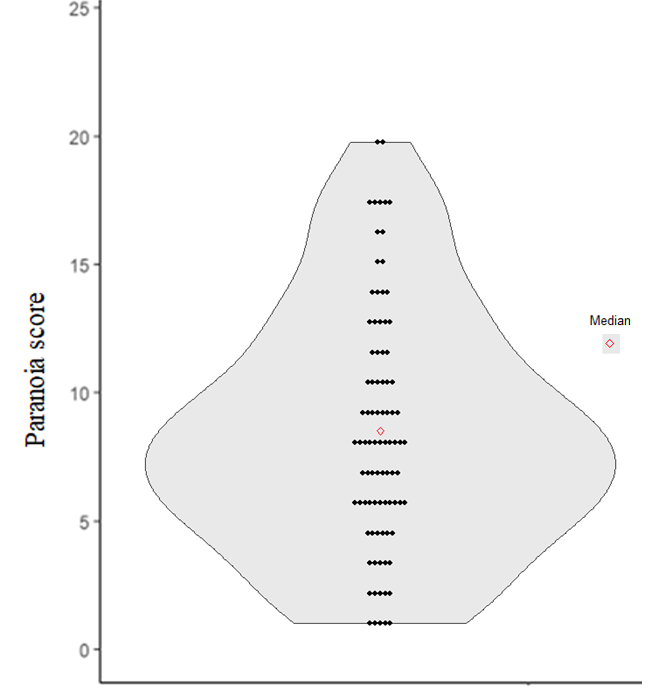
**

**B. Distribution of the score on each PANSS item of the composite paranoia score (range 1-7)**

**
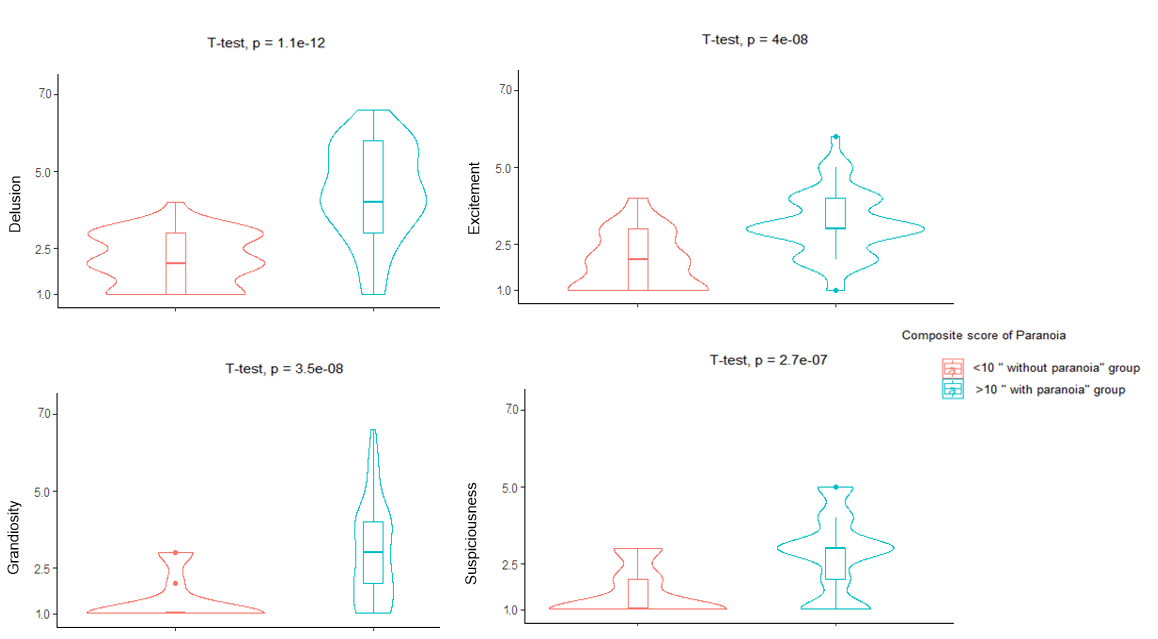
**

**C. Correlation Table**

**parameter1**  **parameter2 rho p.value**

--------------------------------------------------------------------------------------------

Composite PANSS score PANSS_Positive score 0.8586 <0.001

Composite PANSS score PANSS_Negative score 0.0681 0.526

---------------------------------------------------------------------------------------------

*This table summarizes the correlation between the composite paranoia score and the positive PANSS score and the negative PANSS score*

**Supplement 2:** Location of the regions of interest.


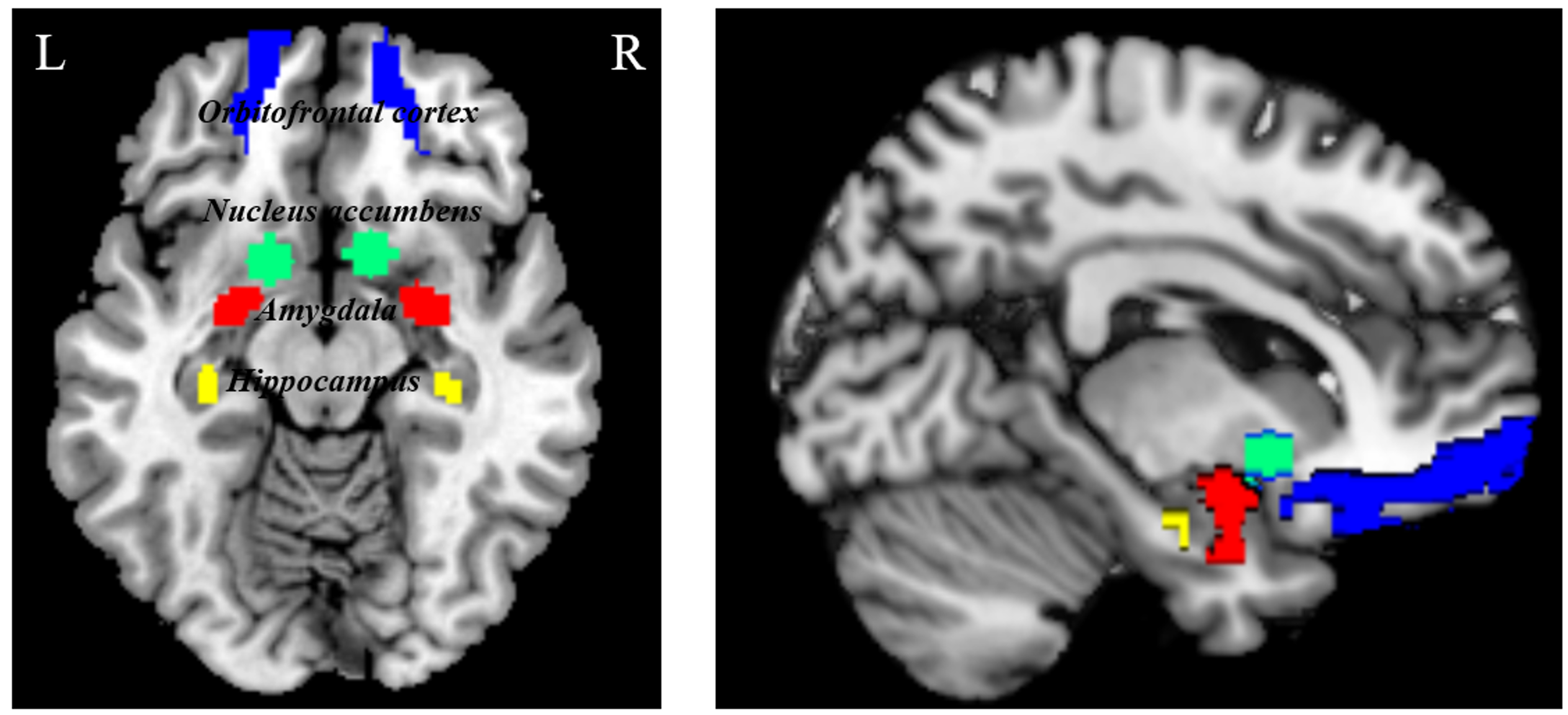


**Supplement 3: Alternative classification method using the P6 item (Method and results)**

1. **Methods**

As an alternative method, we used the patients score on the P6 item of the PANSS to evaluate paranoia. We split our sample into patients with paranoia (P6>= 3) and those without paranoia (< 3). The distribution of the P6 score in the patients is reported in the following figure. Using this score, 39 patients are classified in the paranoia group and 50 in the non-paranoia group.

**
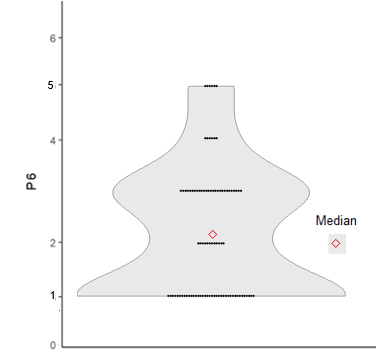

Figure** Distribution of the P6 score in the 89 patients.

1. **Results**

Age, mean FD, medication and ICV were used as covariates of non-interest.

ROI-to-ROI analysis between groups

There are no significant difference in the ROI-to-ROI analysis between the patients with and without paranoia when the cut-off criteria is the P6 item.

ROI-to-voxels analysis between groups

There was a statistically significant increase of the rs-fc in paranoia patients compared to patients without paranoia between the right sup OFC and the left BA 6 and left BA 44 and a statistically significant decrease of the rs-fc between the right sup OFC and the left BA 7 and right BA10 (Table 3).

| **ROI** | **Cluster peak (x, y, z)** | **BA** | **size** | **Cluster p-FWE** | **Cluster p-qFDR** |
| --- | --- | --- | --- | --- | --- |
| ***Increased in paranoia patients*** *compared to patients without paranoia* | | | | | |
| Right sup OFC | -52 8 +38 | BA 6 | 186 | 0.013 | 0.006 |
| Right sup OFC | -50 +14 +8 | BA 44 | 169 | 0.024 | 0.007 |
| ***Decreased in paranoia patients compared to patients without paranoia*** | | | | | |
| Right sup OFC | -08 -74 +38 | BA 7 | 186 | 0.013 | 0.006 |
| Right sup OFC | +16 +44 -04 | BA 10 | 132 | 0.08 | 0.02 |

**Table 3** ROI-to-voxels rs-fc differences between paranoia and non-paranoia groups table. Hippo: Hippocampus, R: right, L: left

ROI-to-ROI association with paranoia severity

There are no significant difference in the ROI-to-ROI analysis when associated with the severity of paranoia as measured by P6 score.

ROI-to-voxels association with paranoia severity

The association between paranoia severity (P6) and the rs-fc connectivity between every ROI and the rest of the brain was evaluated. There were negative correlations suggesting that higher paranoia scores are associated with decreased connectivity between the right amydgala and the right BA 22 and right hippocampus and right BA 39 and BA47.

| **ROI** | **Cluster peak (x, y, z)** | **BA** | **size** | **Cluster p-FWE** | **Cluster p-qFDR** |
| --- | --- | --- | --- | --- | --- |
| Right amydgala | *Negative correlation* | | | | |
|  | +64 -32 +02 | BA 22 | 183 | 0.01 | 0.01 |
| Right hippocampus | *Negative correlation* | | | | |
|  | -50 -62 +22 | BA 39 | 198 | 0.03 | 0.017 |
|  | +22 +44 +12 | BA 47 | 139 | 0.04 | 0.017 |

**Table 5** ROI-to-voxels rs-fc changes with paranoia severity table.

**Supplement 4 : Whole brain Independent Component Analysis using the composite paranoia score to classify the patients into paranoia and non-paranoia sub-groups
A. Methods**

In addition to the ROI analysis, we also performed whole brain comparisons of the rs-fc between the patients and the healthy controls using whole-brain Independent Component Analyses (ICA) with the CONN toolbox. For each participant the FastICA algorithm extracted 30 components. Then, a self-organizing group-level ICA was used to classify the 30 components based on a clustering procedure that accepts only one component per subject in each cluster. Across these 30 components, five components of interest were identified referring to the following rs-networks: the default mode network (DMN), the salience network (SAL), the dorsal attentional network (DAN), the central executive network (CEN), and the motor network (MN)). We compared the rs-fc within each of these five components using an ANCOVA with two groups and with age, years of education and ICV as covariates of non-interest. We used this method to perform the whole-brain comparison between 1) healthy controls and schizophrenia patients, 2) healthy controls and patients with paranoia. For whole-brain ICAs, we considered the cluster-size p-value qFDR corrected < 0.05 to be significant.

**B. Results: Whole-brain ICA to compare rs-fc between patients with schizophrenia and healthy controls**

At a cluster threshold p-qFDR corrected < 0.05, (t(160)> 3.35, limit cluster size (k) = 100), we did not observe any statistically significant difference between healthy controls and patients in the rs-fc of the components exploring the SAL and the CEN. We observed a decrease of the intrinsic rs-fc of the Brodmann area (BA) 23 within the DMN component, a decrease of the intrinsic rs-fc of BA 39 within the DAN component and an increase of the intrinsic rs-fc of the BA 4 within the MN component in patients with schizophrenia compared to healthy controls.

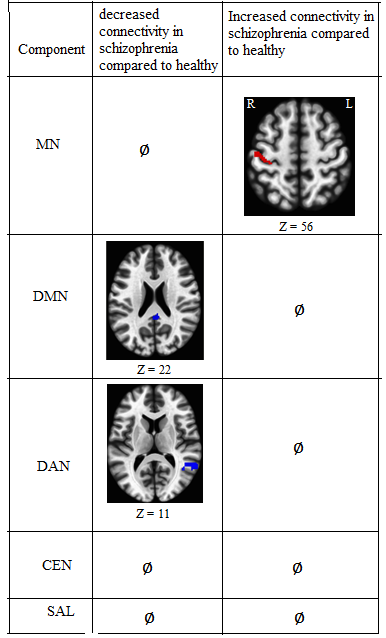

Whole-brain ICA maps to compare rs-fc between patients with schizophrenia and healthy controls. cluster

threshold p-qFDR corrected < 0.05, (t(160)> 3.35, minimum cluster size (k) = 110).

| **Component** | **Cluster peak (x, y, z)** | **BA** | **size** | **Cluster p-FWE** | **Cluster p-qFDR** |
| --- | --- | --- | --- | --- | --- |
| *DMN* | *decreased connectivity in patients with paranoia compared to healthy controls* | | | | |
|  | -04 -38 +28 | BA 23 | 110 | 0.03 | 0.03 |
|  | *increased connectivity in patients with paranoia compared to healthy controls* | | | | |
|  | ø | | | | |
| MN | *decreased connectivity in patients with paranoia compared to healthy controls* | | | | |
|  | ø | | | | |
|  | *increased connectivity in patients with paranoia compared to healthy controls*  -48 -16 52 BA 4 126 0.01 0.01 | | | | |
| DAN | *decreased connectivity in patients with paranoia compared to healthy controls* | | | | |
|  | +42 -52 +16 | BA 39 | 167 | 0.001 | 0.001 |
|  | *increased connectivity in patients with paranoia compared to healthy controls* | | | | |
|  | ø | | | | |

Whole-brain ICA table to compare rs-fc between patients with schizophrenia and healthy controls. cluster threshold p-qFDR corrected < 0.05, (t(160)> 3.35, minimum cluster size (k) = 100). *BA: Brodmann area*

**C. Results: Whole-brain ICA to compare rs-fc between patients with paranoia and healthy controls**

We compared the whole-brain rs-fc between the 49 patients with paranoia and the 76 healthy controls. At a cluster threshold p-qFDR corrected < 0.05, (t(121)> 3.37, limit cluster size (k) = 50), we failed to observe any difference between healthy controls and patients with paranoia in the SAL, DAN, and MN. However, we observed a decrease of the intrinsic rs-fc of the BA 23 within the DMN component, as well as increased intrinsic rs-fc in the BA 8, 9, 21, 39, 46 and 47 within the DMN component along with decreases of the rs-fc in the BA 39 within the CEN component in patients with paranoia compared to healthy controls.

and in the CEN. These difference highlighted a decreased connectivity in patients with paranoia compared to healthy controls bilaterally in the posterior cingulate cortex and in the right parietal cortex and an increased connectivity in patients with paranoia compared to healthy controls bilaterally in the parietal cortex and in the dorsolateral prefrontal cortex (Supplementary Figure 3, Supplementary Table 3).

| **Component** | **Cluster peak (x, y, z)** | **BA** | **size** | **Cluster p-FWE** | **Cluster p-qFDR** |
| --- | --- | --- | --- | --- | --- |
| *DMN* | *decreased connectivity in patients with paranoia compared to healthy controls* | | | | |
|  | -04 -45 +20 | BA 23 | 78 | 0.041 | 0.009 |
|  | *increased connectivity in patients with paranoia compared to healthy controls* | | | | |
|  | -42 +20 +42 | BA 8 | 133 | 0.21 | <0.001 |
|  | +44 +38 -04 | BA 47 | 104 | 0.94 | 0.003 |
|  | +48 -54 +50 | BA 39 | 94 | 0.017 | 0.004 |
|  | -48 +40 +04 | BA 46 | 185 | 0.18 | <0.001 |
|  | -58 -52 +38 | BA 39 | 153 | 0.79 | <0.001 |
|  | +56 -38 -16 | BA 21 | 67 | 0.079 | 0.015 |
|  | +52 +26 +22 | BA 9 | 50 | 0.22 | 0.04 |
|  |  |  |  |  |  |
| *CEN* | *decreased connectivity in patients with paranoia compared to healthy controls* | | | | |
|  | +42 -52 +16 | BA 39 | 331 | <0.001 | <0.001 |
|  | *increased connectivity in patients with paranoia compared to healthy controls* | | | | |
|  | ø | | | | |
| DAN | *decreased connectivity in patients with paranoia compared to healthy controls* | | | | |
|  | ø | | | | |
|  | *increased connectivity in patients with paranoia compared to healthy controls* | | | | |
|  | ø | | | | |
| SAL | *decreased connectivity in patients with paranoia compared to healthy controls* | | | | |
|  | ø | | | | |
|  | *increased connectivity in patients with paranoia compared to healthy controls* | | | | |
|  | ø | | | | |
|  |  |  |  |  |  |
| MN | *decreased connectivity in patients with paranoia compared to healthy controls* | | | | |
|  | ø | | | | |
|  | *increased connectivity in patients with paranoia compared to healthy controls* | | | | |
|  | ø | | | | |

Supplementary Table 3: Whole-brain ICA table to compare rs-fc between patients with paranoia and healthy controls. cluster threshold p-qFDR corrected < 0.05, (t(121)> 3.37, limit cluster size (k) = 50). *BA: Brodmann area*


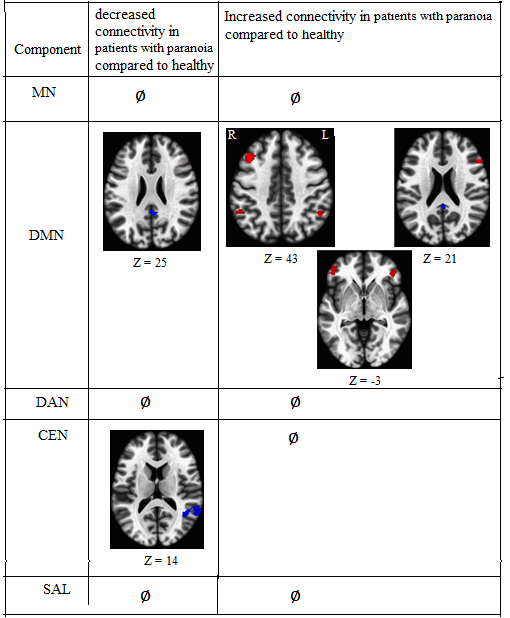

Supplementary figure 3: Whole-brain ICA map to compare rs-fc between patients with paranoia and healthy controls

**Supplement 5.** **Additional analysis to** **reassure that motion differences between groups did not have an impact on rs-fc analyses.**

1. **Absolute displacement**

In addition to the preprocessing steps performed to insure that the micro movements of the participants are taken into account in the analyses and did not drive the main effect of the present study, the motion parameters of the participants are displayed in Supplementary Figure 4.
Participants exclusion threshold: absolute head motion involved translation greater than 2 mm or rotation greater than 2°. In addition, we performed ANOVAs to compare the displacement in the 6 directions (3 translations and 3 rotations) between the 3 groups. These analyses demonstrated that our dataset contained no strong movements that cannot be accounted for using the preprocessing steps. Moreover, even if the SCZ patients tend to present higher amplitude ranges in the displacement than the healthy controls, there is no statistically significant difference in the Mean or the range of displacement across the 3 groups (Supplementary Table 4).

1. **Relative displacement**

Participants exclusion threshold: mean FD > 0.5mm. See Supplentary Figure 5 and Supplementary Table 5 for group comparison. Ni significant difference where observed between the 3 groups mean FD. We still used mean FD as a covariate of non interest in all further analyses.

|  |  | **Patients with Paranoia** | **Patients without Paranoia** | **Healthy** | **ANOVA** | **Multiple comparison correction (P<0.004)** | | | | **Post hoc (tukey method) provided if p value survives multiple comparison correction** |  |
| --- | --- | --- | --- | --- | --- | --- | --- | --- | --- | --- | --- |
| tx | mean | 0.0003 | -0.0407 | -0,0157 | F(2)=0.286, p=0.75 | | / | / | | |  |
|  | sd | 0.6769 | 0.1908 | 0,1790 | F(2)=2.485, p=0.09 | | / | / | | |  |
| ty | mean | 0.0761 | 0.0350 | 0,0649 | F(2)=0.1426, p=0.86 | | / | / | | |  |
|  | sd | 0.1556 | 0.1377 | 0,0966 | F(2)=4.301, p=0.02 | | / | / | | |  |
| tz | mean | -0.0304 | 0.3019 | 0,1090 | F(2)=3.617, p=0.03 | | / | / | | |  |
|  | sd | 0.3152 | 0.3202 | 0,1782 | F(2)=4.847, p=0.009 | | / | / | | |  |
| rx | mean | 0.0014 | 0.0038 | 0,0009 | F(2)=1.54, p=0.21 | | / | / | | |  |
|  | sd | 0.0031 | 0.0042 | 0,0030 | F(2)=1.98, p=0.14 | | / | / | | |  |
| ry | mean | -0.0001 | -0.0003 | -0,0003 | F(2)=0.057, p=0.94 | | / | | / | |  |
|  | sd | 0.0028 | 0.0015 | 0,0015 | F(2)=2.823, p=0.06 | | / | | / | |  |
| rz | mean | -0.0008 | 0.0003 | 0,0002 | F(2)=0.282, p=0.75 | | / | | / | |  |
|  | sd | 0.0052 | 0.0026 | 0,0016 | F(2)=2.963, p=0.055 | | / | | / | |  |

**Supplementary Table 4**: Between group comparison of the mean and sd for each of the 6 directions (translation x (tx), translation y (ty), translation z (tz), rotation x(rx), rotation y (ry), rotation z(rz)).

|  |  | **Patients with Paranoia** | **Patients without Paranoia** | **Healthy** | **ANOVA** |
| --- | --- | --- | --- | --- | --- |
| Mean FD | mean | 0.175 | 0.1813 | 0.1578 | F(2)=1.051, p=0.352 |

**Supplementary Table 5**: Between group comparison of the mean Frame wise displacement (FD).


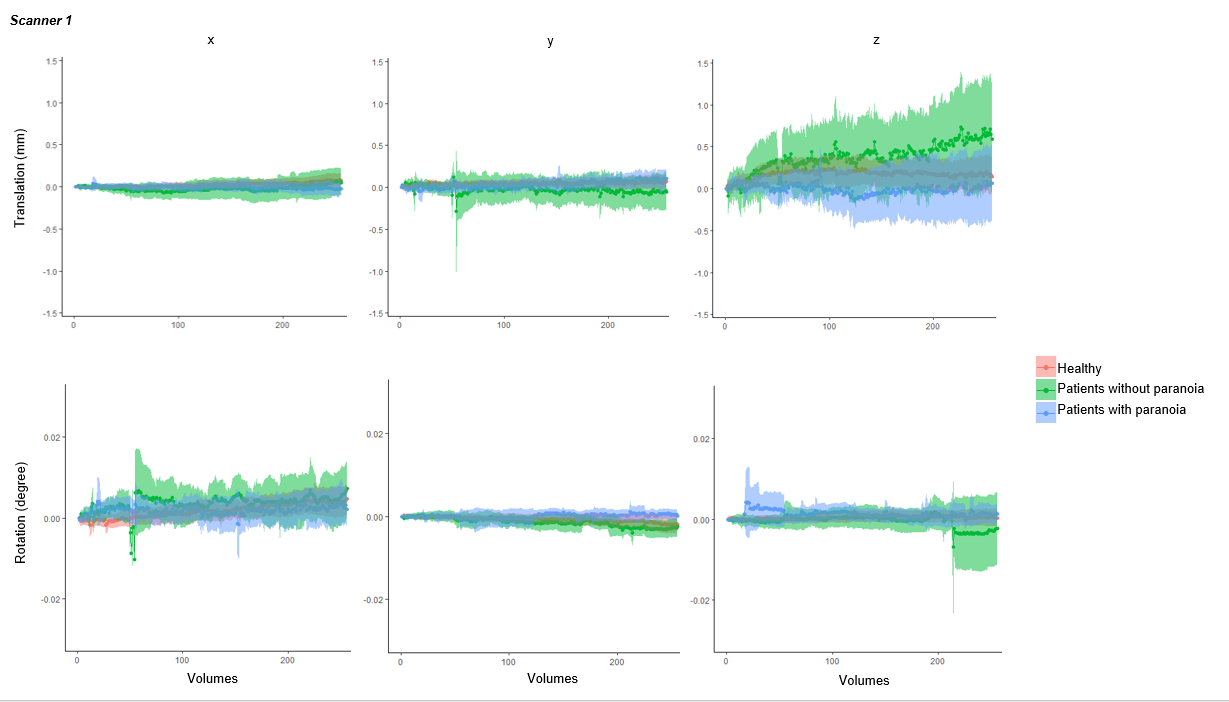


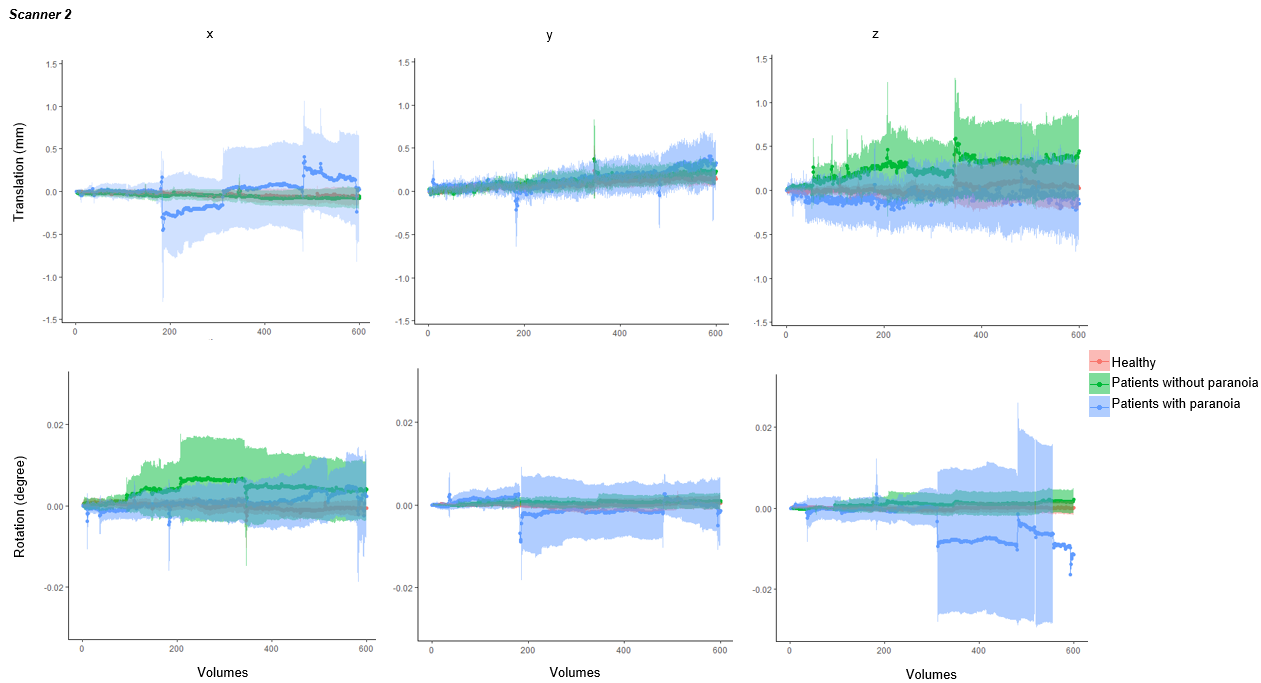


**Supplementary Figure 4: Plot of the head motion in each of the 6 directions** (Mean+CI at each time point), for each subgroup, in each scanner

**Supplementary Figure 5. Plot of the mean FD across the 3 groups**

**
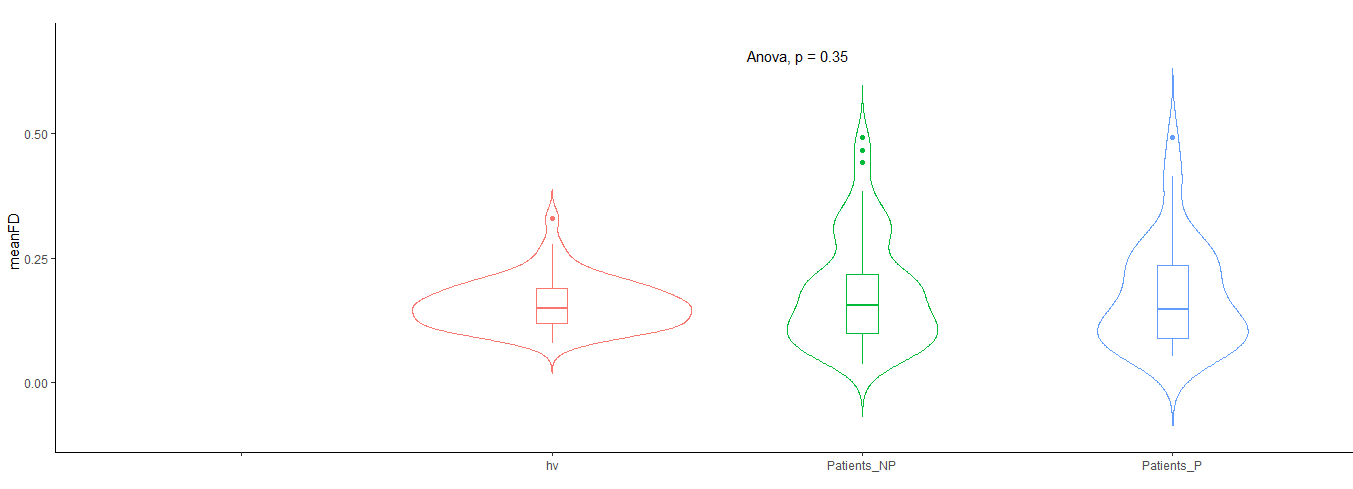
**

**Supplementary Figure 5. Plot of the mean FD across the 3 groups.** Violin plot and box plot of the Mean FD repartition in the individuals across the 3 groups.

**Supplementary Figure 6: comparison for to ROI-to-ROI rs-fc between the two scanners for each subgroup.**

**
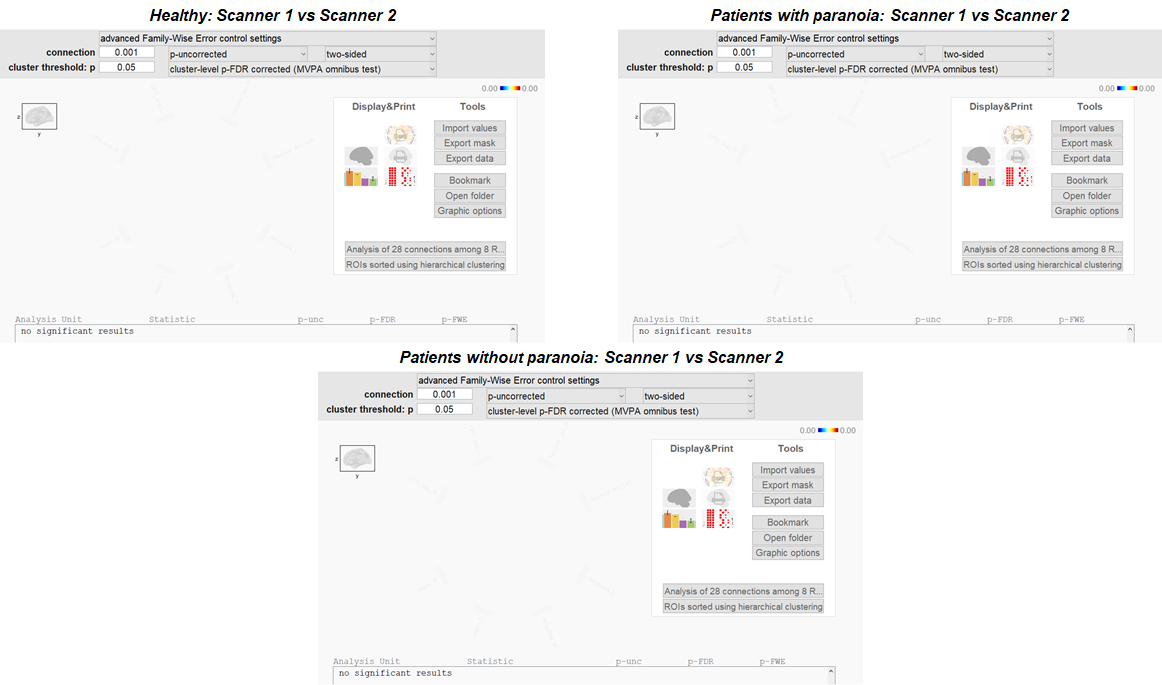
**
